# Prolonged Activation of Invariant Natural Killer T Cells and T_H_2-Skewed Immunity in Stroke Patients

**DOI:** 10.3389/fneur.2017.00006

**Published:** 2017-01-19

**Authors:** Connie H. Y. Wong, Craig N. Jenne, Patrick P. Tam, Caroline Léger, Andres Venegas, Karla Ryckborst, Michael D. Hill, Paul Kubes

**Affiliations:** ^1^Centre for Inflammatory Diseases, Department of Medicine, School of Clinical Science, Monash University, Melbourne, VIC, Australia; ^2^Department of Physiology and Pharmacology, Snyder Institute for Chronic Diseases, University of Calgary, Calgary, AB, Canada; ^3^Department of Critical Care, Snyder Institute for Critical Care, University of Calgary, Calgary, AB, Canada; ^4^Stroke Unit, Department of Clinical Neurosciences, Hotchkiss Brain Institute, University of Calgary, Calgary, AB, Canada

**Keywords:** stroke, immune suppression, infection, IL-10, *i*NKT cells

## Abstract

**Background:**

Infection is highly prevalent and contribute significantly to mortality of stroke patients. In addition to the well described robust systemic lymphocytopenia and skewed T helper 2 (T_H_2)-immunity after stroke, emerging experimental evidence demonstrate that the development of infection poststroke is attributed by the activation of invariant natural killer T (*i*NKT) cells. In this prospective study, we examined the levels of a broad spectrum of inflammatory mediators, the activation status of *i*NKT cell in the blood of patients with various degree of stroke severity, and investigate whether these parameters differ in patients who later develop poststroke infections.

**Methods and results:**

We obtained blood from stroke patients and matching controls to perform flow cytometry and multiplex measurement of inflammatory mediators. Our data suggest a pronounced activation of *i*NKT cells in stroke patients as compared with matched Healthy and Hospital control patients. The magnitude of *i*NKT activation is positively correlated with the severity of stroke, supporting the hypothesis that *i*NKT cells may contribute in the modulation of the host immune response after stroke-associated brain injury. In addition, stroke severity is closely correlated with decreased T_H_1/T_H_2 ratio, increased production of interleukin (IL)-10, with infected stroke patients showing exacerbated production of IL-10.

**Conclusion:**

Stroke triggers a robust and sustained shift in systemic immunity in patients, including specific lymphopenia, robust activation of *i*NKT cells, systemic production of IL-10, and a prolonged T_H_2-skewed immunity, all are potential contributors to severe immune suppression seen in patients after stroke. Future studies with large sample size will provide potential causality relationship insights.

## Introduction

Ischemic stroke is a common and debilitating cerebrovascular event that is caused by the sudden impairment of blood flow to regions of the brain. Up to 65% of stroke patients develop infection (1), and more than 30% die as a direct result (2), making infection a highly relevant clinical problem. Increased bacterial infections in stroke patients suggest that the immune response, which normally is responsible for eliminating these infections in healthy individuals, is impaired after ischemic brain injury. Emerging evidence now indicates that stroke induces profound systemic immune effects (3), including severe reductions in the number of circulating lymphocytes and altered lymphocyte and monocyte function (4, 5). Despite accumulating evidence supporting the notion of a stroke-induced peripheral anti-inflammatory state in response to the overwhelming cerebral inflammation following stroke (6), the molecular mechanisms resulting in systemic immune suppression after stroke in humans have, for the most part, remained elusive.

The balance between pro- and anti-inflammatory cytokines determines the proficiency of the immunological response and has the potential to influence both the fate of the injured brain and the threshold to developing complications such as infection. In the clinical setting of stroke, the balance between pro- and anti-inflammatory cytokines is an important prognostic factor (6). Stroke patients were found to present with a rapid increase in plasma cytokines resulting in a low ratio of pro-inflammatory tumor necrosis factor alpha (TNFα) to anti-inflammatory interleukin (IL)-10, preceding the appearance of infection (7). This observation supports the hypothesis of stroke-induced immunosuppression *via* a decrease in systemic T_H_1/T_H_2 ratio. Surprisingly, poststroke T cell priming to an increased IL-4 expression following mitogenic stimulation was evident in the peripheral blood of patients in the post-acute phase of stroke (8). Despite this, it is important to note of the timing of this apparent immune shift following stroke. Generally, lymphocytes and the adaptive immune response could be expected to control infection 5–7 days after stroke, dysfunction in these cells does not explain the observed increase in infection in the first 3 days (9). In addition, the development of infection in the mouse model of stroke takes only a few hours (10). This difference in the temporal development of a T_H_2-skewed immune response following stroke suggests the shift must be orchestrated by mechanisms distinct from the conventional adaptive immune response.

In our previous work with an experimental model of ischemic stroke, we demonstrated that invariant natural killer T (*i*NKT) cells play an important role in regulating poststroke immunosuppression and infections (10). *i*NKT cells are a distinct lymphocyte lineage that can rapidly produce large quantities of both T_H_1 [interferon gamma (IFNγ), TNFα] and T_H_2 (IL-4, IL-10) cytokines, giving these cells a unique ability to have wide-ranging roles in the regulation of immunity (11). Despite their location in a site remote from the brain, we showed that circulating *i*NKT cells and *i*NKT cells resident in the liver were able to rapidly respond to ischemic stroke, and release predominantly a T_H_2-type cytokine IL-10, rendering the host more susceptible to bacterial infections (10). This intriguing finding highlighted a connection between brain injury sustained following ischemic stroke and robust functional impairment of peripheral immune cells, in particularly, *i*NKT cells. In this prospective clinical study, we aimed to delineate the systemic immune profile of stroke patients over time. Specifically, we conducted a matched cohort study to characterize the temporal T_H_1/T_H_2 cytokine, chemokine, and *i*NKT cell response in the blood of stroke patients up to 3 months after stroke onset. In addition, we sought to explore whether an association exists between stroke severity with the degree of peripheral *i*NKT cell activation and T_H_2-skewed systemic immunity.

## Materials and Methods

### Study Design

This study was approved by the Conjoint Health Research Ethics Board (CHREB) at the University of Calgary. This was a prospective cohort study designed to understand the influence of stroke on the immune system. We enrolled patients with arterial ischemic stroke in the Stroke clinic of the Foothills Medical Center. Radiologically confirmed stroke patients with an National Institute of Health Stroke Scale (NHISS) score of 4–24 were enrolled within 24 h of admission after providing written informed consent for participation in the study. All of the medications given to the patients after stroke were documented. Patients with known chronic inflammatory or infectious diseases (e.g., rheumatoid arthritis, lupus, Crohn’s disease, etc.), cancer, hematological diseases, or severe renal or liver failure, as well as those who were under treatment with anti-inflammatory drugs, were excluded. In addition, patients with known immunosuppressive diseases (including diabetes and human immunodeficiency virus infection), a known history of low neutrophil (<2 × 10^6^/mL) or lymphocyte (<1 × 10^6^/mL) counts, on immunosuppressive drug, asplenia or asplenism, as well as those who were under treatment with antibiotics or any forms of adrenergic receptor antagonists, were also excluded.

Following consent, blood was collected from each patient into lithium-heparin-containing vacutainer tubes (CIE) as soon as possible after admission and on days 1, 2, 7 (or discharge, D/C) and 90 days thereafter. We also collected demographic (age, sex, medical history, medications), admission (presenting illness, vital signs, routine laboratory results, initial antimicrobials, fluids), microbiology, stroke severity (NIHSS), and physiologic (ventilatory support, vasoactive agents, renal function, and liver function) information. From the start of enrollment, the stroke patients were observed for the development of spontaneous infections, including but not limited to pneumonia, urinary tract infections, and cellulitis.

Two control groups were included. The first control group (Hospital control) consisted of nine patients admitted to the clinic for stroke-like symptoms but failed to meet clinical criteria for stroke and have no known diseases. The second control group (Healthy group) consisted of 10 healthy individuals recruited for a one time blood donation. Both controls groups were sex and age matched (10-year-spanning categories). The same inclusion criteria as stated for stroke patients applied to both groups of controls.

### Sample Collection

Sample collection and analyses in the different groups of patients and controls were carried out under the same conditions and within the same time frame. Following patient consent, blood was collected within 24 h of stroke onset to give the “Admission” time point, and then at 1, 2, 7 (or at discharge), and 90 days after admission. Blood was collected into lithium-heparin-containing vacutainer tubes (CIE) and divided into three portions − (1) routine hospital microbiology culture, (2) immune cell analysis, and (3) measurement of inflammatory mediator levels. Blood collected for microbiology was immediately sent to Calgary Laboratory Services (CLS) Microbiology at the Foothills Medical Center, Canada. Blood collected for immune cell analysis was immediately sent to the laboratory for processing and fluorescence-activated cell sorting (FACS). Blood collected for measurement of inflammatory mediator levels was immediately centrifuged at 1,200 *g* for 15 min in a swinging bucket centrifuge. The resultant plasma was collected and aliquoted for storage at −80°C until inflammatory mediator analyses could be performed.

### Hospital Microbiology Culture Analysis

Clinical bacteriological analysis was performed by CLS Microbiology at the Foothills Medical Center, Canada. For blood culture, venipuncture was the technique of choice for obtaining blood. Blood culture bottles (green for aerobic and orange for anaerobic) were used for collection. The bottle tops were disinfected with 70% isopropanol and allow to dry for 1 min. After selecting blood collection site, the site was scrubbed with 70% isopropanol for a minimum of 30 s and allowed to dry. Using 10% PVP Iodine or Chlorhexidine gluconate solution, the site was cleansed for 30 s using a circular motion, starting at the center of the site and moving outward. The site was allowed to dry for a minimum of 1 min. After blood collection, the bottles were gently mixed by inversion to prevent clotting. Residual iodine was removed from the skin with 70% isopropanol after venipuncture. Sputum and urine samples were collected in sterile containers. All collection tubes and containers were labeled with the required patient identifiers, kept at room temperature, and processed within 4 h of collection.

### Immune Cell Analysis

Collected blood was processed and analyzed by FACS within 2 h of collection. A small aliquot of the blood (10 µL) was used to perform cell count with a hemocytometer. The remainder of the blood (approximately 3 mL) was diluted at a 1:1 ratio with saline and carefully layered onto 5 mL of Lymphoprep (Axis-Shield, Norway). Gradients were resolved through centrifugation for 20 mins at 400 *g* in a bench-top centrifuge with no brake, after which the interface containing the lymphocytes was collected and washed in cold FACS wash buffer (FWB; PBS, 2% fetal calf serum, 0.5 mM EDTA). For analysis of leukocyte populations, single cell suspensions of peripheral blood from subjects were stained with PE-conjugated PB57-loaded CD1d-tetramer (NIH Tetrameter Facility, Atlanta, GA, USA), PerCP-conjugated anti-CD3 (OKT3, eBioscience), Alexa Fluor 450-conjugated anti-CD4 (OKT4, eBioscience), and FITC-conjugated anti-CD69 (FN50, eBioscience). All samples were analysed on an Attune acoustic focusing cytometer (Applied Biosystems).

### Measurement of Inflammatory Mediator Levels

Quantification of the concentrations of inflammatory mediators was performed using the validated Luminex bead-based multiplexing assay from R&D Systems (Minneapolis, MN, USA) according to manufacturer’s instructions (12). Briefly, plasma samples were thawed quickly at 37°C, centrifuged at 20,800 *g* at 4°C for 10 min and then stored on ice. Plasma samples were diluted as recommended by the manufacturer and a total of 25 µL of diluted plasma were distributed to individual wells on a 96-well plate each containing capture bead cocktail. The plate was then incubated, in the dark, for 2 h at room temperature on a plate shaker set at 500 rotations per minute (rpm). Wells were washed three times with 100 µL of wash buffer, and then 25 µL of diluted biotin antibody cocktail was added. The plate was subsequently incubated, in the dark, for 1 h at room temperature on a plate shaker set at 500 rpm. Wells were washed three times with 100 µL of wash buffer, and 25 µL of diluted Streptavidin-PE was added to each well. The plate was incubated for 30 min at room temperature on a plate shaker set at 500 rpm. Wells were washed three times with 100 µL of wash buffer, resuspended in 75 µL of wash buffer, and the plate was read using a Luminex 200 apparatus (Applied Cytometry Systems, UK). The data were analyzed using the StarStation V.2.3 (Applied Cytometry Systems, UK).

### Statistical Analyses

All values were expressed as mean ± SEM. Parametric and non-parametric statistical tests were used for the analyses. Data were compared either by unpaired two-tailed Student’s *t*-test and one-way ANOVA with Bonferroni multiple comparisons *post hoc* test or unpaired two-tailed Mann–Whitney *U* test and Kruskal–Wallis test. Pearson’s correlation coefficient (*r*) was used to measure the strength of linear relationship between two variables, with 95% confidence interval expressed in brackets. We considered *p*-values <0.05 statistically significant.

## Results

A total of 36 stroke patients were enrolled in the study, 7 of whom were unable to be contacted or deceased at the 90 days time point. The baseline characteristics and clinical outcomes of the 36 enrolled patients and their age-matched 9 Hospital and 10 Healthy controls were reported previously (13) and detailed here in Table 1. We examined the presence and identity of pathogens in specimens such as blood, urine, and sputum in a prospective manner with strict inclusion/exclusion patient enrollment criteria. In this study, 8 of the 36 patients enrolled (22.2%) showed positive cultures in blood, urine, or sputum by day 7 of stroke onset. No culturable organisms were found in the blood, urine, or sputum at any time point of Healthy and Hospital control patients using the same technique. The identity of pathogens found in the specimens of the infected stroke patients was examined and reported (13). The relatively small patient sample size due to strict inclusion/exclusion patient enrollment criteria in this study indeed does not represent the population norm, but the infection incidence is within the range of those reported (14).

**Table 1:** **Baseline characteristics of the enrolled subjects**.

Characteristics	Stroke without infection	Stroke with infection	Hospital	Healthy
Number of patients, no. (%)	28 (77.8)	8 (22.2)	0 (0)	0 (0)
Age, years, median (IQR)	65.5 (56.5, 72.5)	76.5 (72.75, 83.5)	71.0 (47.0, 83.0)	63.0 (43.75, 79.5)
Male sex, no. (%)	16 (57.1)	6 (75)	5 (55.6)	5 (50%)
Mortality, no. (%)	2 (7.1)	3 (37.5)	0 (0)	0 (0)
National Institute of Health Stroke Scale, median (IQR)	6.5 (4, 10.75)	8 (4.25, 20.5)	–	–
Localization of infarction, no. (%)				
MCA	16 (57.1)	6 (75)	–	–
ACA	1 (3.6)	0 (0)	–	–
PCA	3 (10.7)	0 (0)	–	–
Cerebella	1 (3.6)	0 (0)	–	–
Pontine/midbrain	4 (14.3)	0 (0)	–	–
Multiple territories	3 (10.7)	2 (25)	–	–
Hemispheric, no. (%)				
Left	16 (57.1)	4 (50)	–	–
Right	8 (28.6)	1 (12.5)	–	–
Bilateral	0 (0)	2 (25)	–	–
Stem	3 (10.7)	0 (0)	–	–
Shower	1 (3.6)	1 (12.5)	–	–
Infection site, no. (%)				
Chest	–	2 (25)	–	–
Urinary tract	–	6 (75)	–	–
Mortality from infection, no. (%)				
Chest	–	2 (100)	–	–
Urinary tract	–	1 (16.7)	–	–

*The National Institute of Health Stroke Scale is composed of 11 items to use as a tool by clinicians to objectively quantify the stroke impairment*.

*ACA, anterior cerebral artery; MCA, middle cerebral artery; PCA, posterior cerebral artery; IQR, interquartile range*.

**Figure 1:**
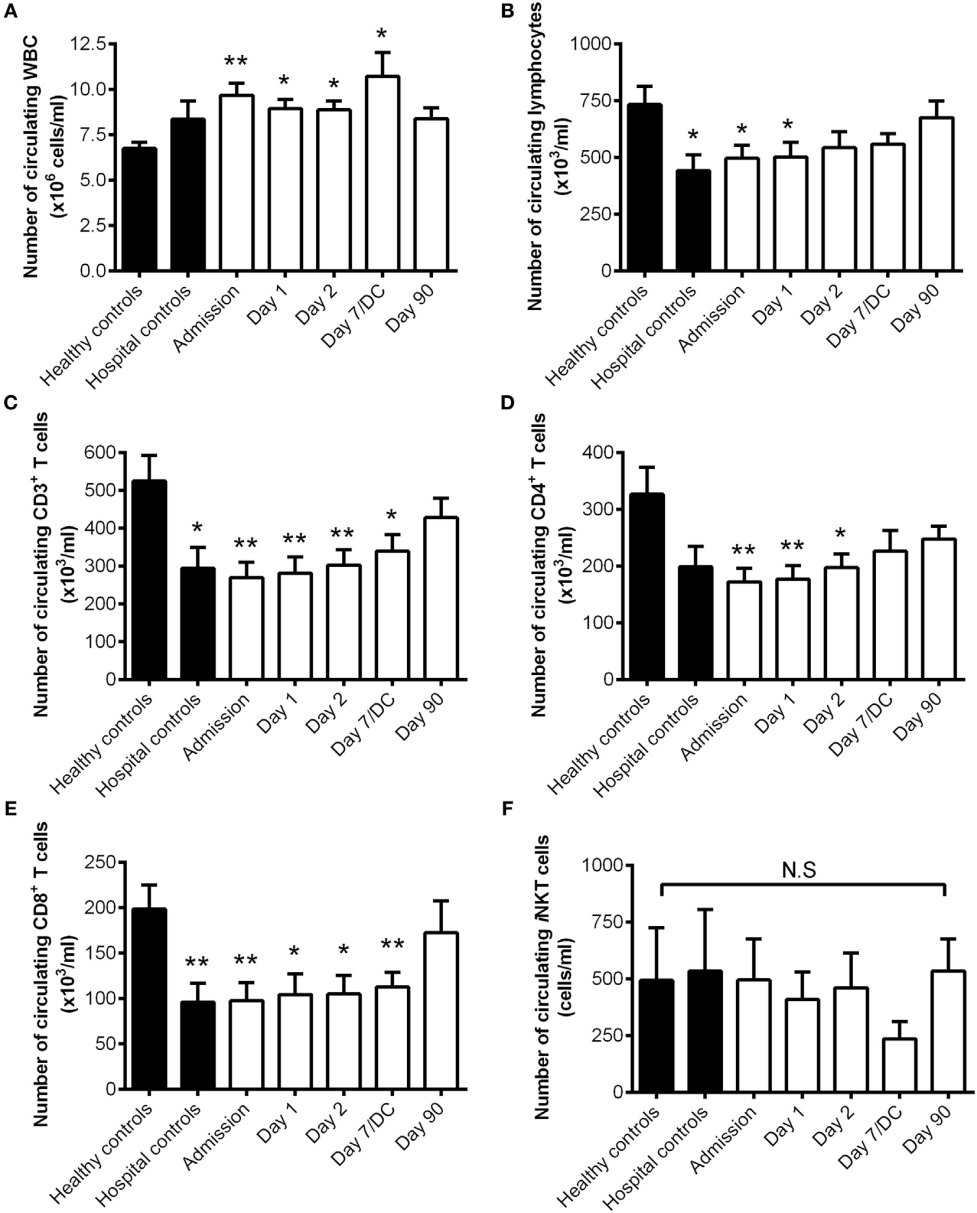
**The number of circulating invariant natural killer T (*i*NKT) cells unchanged after stroke**. The number of circulating white blood cells [WBC; **(A)**], lymphocytes **(B)**, CD3^+^ T cells **(C)**, CD4^+^ T cells **(D)**, CD8^+^ T cells **(E)**, and *i*NKT cells **(F)** were quantified in Healthy (*n* = 10) and Hospital (*n* = 9) controls as well as stroke patients at Admission (*n* = 36) and 1 day (*n* = 36), 2 days (*n* = 36), 7 days (or at discharge, DC; *n* = 14), and 90 days (*n* = 27) after stroke onset. Error bars, SEM. *p < 0.05, **p < 0.01 vs Healthy controls unpaired two-tailed Student’s *t*-test. N.S. denotes no statistical significance.

We first examined the circulating white blood cell counts in stroke patients compare to their Healthy and Hospital controls. There was a significant increase in the number of circulating leukocytes in patients with stroke at Admission and up to 7 days following stroke onset compared to their Healthy controls (Figure 1A). Despite this, stroke patients had a significant but transient reduction in the number of circulating lymphocytes at Admission and on day 1 after stroke onset as compared to matched Healthy controls (Figure 1B). This reduction was contributed by the loss of T lymphocytes (Figure 1C), in both CD4^+^ T cells (Figure 1D) and CD8^+^ T cells (Figure 1E). Unlike the conventional T lymphocyte population, stroke injury did not reduce the number of *i*NKT cells in the blood and this population of specialized immune cells also did not decrease in numbers during the study period (Figure 1F). Intriguingly, the numbers of lymphocytes, T cells, particularly CD8^+^ T cells were similarly decreased in Hospital controls compared with Healthy controls. This suggests a non-specific stress response affecting circulating lymphocyte numbers in hospitalized patients that is not related to the stroke injury or contributing to the host susceptibility to infection. Lymphocytopenia is a common finding in hospital patients (15), this is in spite of rapid enrollment of this group of patients to the study as they waited at the Emergency department. Nevertheless, we found lymphocyte counts were equally reduced in stroke patients with and without infection and irrespective of stroke severity in this study.

**Figure 2:**
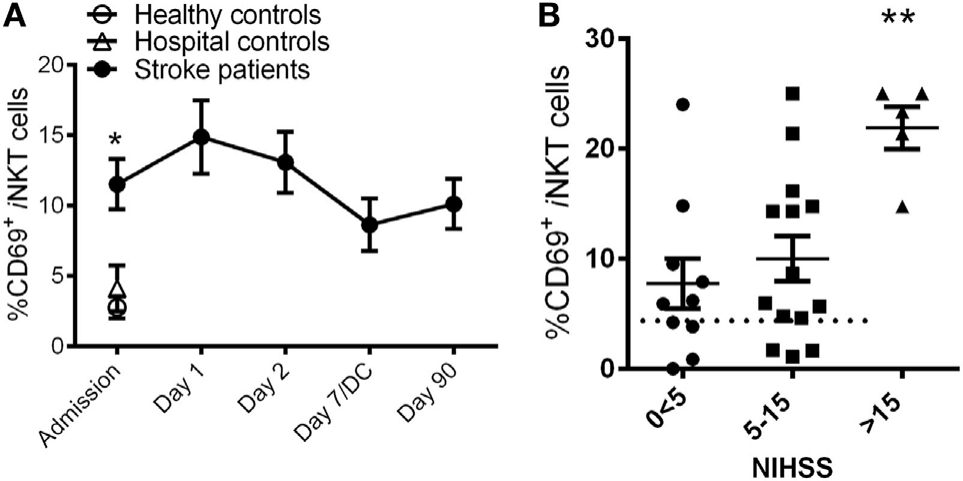
**Rapid invariant natural killer T (*i*NKT) cell activation after stroke**. **(A)** The expression of CD69 in *i*NKT cells was quantified in Healthy (*n* = 10) and Hospital (*n* = 9) controls as well as stroke patients at Admission (*n* = 36) and 1 day (*n* = 36), 2 days (*n* = 36), 7 days (or at discharge, DC; *n* = 14), and 90 days (*n* = 27) after stroke onset. Error bars, SEM. *p < 0.05 vs Healthy controls, unpaired two-tailed Student’s *t*-test. The activation of *i*NKT cells of stroke patients with various National Institute of Health Stroke Scale (NIHSS) at Admission was also measured **(B)**. Error bars, SEM. **p < 0.01 one-way ANOVA with Bonferroni multiple comparisons *post hoc* test.

We next examined if peripheral *i*NKT cells are activated in stroke patients. Both Healthy and Hospital patient controls showed basal *i*NKT cells activation, whereas stroke patients demonstrated an approximately threefold increase of *i*NKT cell activation (Figure 2A). It is important to note that we did not stimulate the cells with mitogens, we are reporting the activation status of the *i*NKT cells as they are isolated from the circulating blood of the patients. This stroke-induced activation of *i*NKT cells was observed for up to 3 months following the stroke event (Figure 2A). The degree of *i*NKT cell activation at Admission in patients with stroke was positively correlated to stroke severity such that patients with higher NIHSS > 15 at the onset of stroke demonstrated significantly greater activation of *i*NKT cells compared to patients presented with NIHSS of less than 15 (Figure 2B).

To examine the specific effect of stroke on the host systemic immune system over time, we measured the plasma levels of 55 cytokines, chemokines, and other markers of inflammation and calculated the average fold changes between stroke patients and their matching Hospital controls over the 3 months enrollment period. We have chosen to compare the levels of inflammatory markers of stroke patients with Hospital controls rather than the Healthy cohort to examine stroke-specific response and not merely stress associated with hospital admission. In addition, we have summarized the results of each inflammatory markers in the tabular format instead of showing 55 graphs to provide a snapshot summary of temporal changes. The fold changes are detailed in Table 2, with the plasma level of inflammation markers at Admission listed in an ascending order. In addition, we have marked the cells in Table 2 with different colors according to the amount of fold change relatively to the Hospital control group. Red and pink cells denote the level of inflammatory marker in the plasma that was lowered in the stroke patients compared to Hospital controls by more than 2-fold and 1.5-fold, respectively. In contrast, navy and light blue cells denotes the level of inflammatory marker in the plasma that was elevated in the stroke patients compared to Hospital controls by more than 2 and 1.5 folds, respectively. Overall, there were a greater number of inflammatory markers that showed twofold or higher increase in production after stroke.

**Table 2:** **Fold changes of plasma cytokines and chemokines in stroke patients as compared to Hospital controls**.

	Admission	Day 1	Day 2	Day 7/DC	Day 90
Interleukin (IL)-12p40	0.2038	0.3217	0.1675	0.1475	0.3312
IL-6	0.5368	6.7618	0.8140	0.8401	0.3000
IFNα2	0.7338	0.6131	0.6308	0.5549	0.9889
MIG	0.7614	0.6714	0.9917	0.7227	1.0008
IL-16	0.7943	0.7663	0.7629	0.6924	0.9124
IL-3	0.8090	0.6260	0.5930	0.7428	0.5605
β-NGF	0.8315	0.7193	0.8044	0.7851	5.7711
IL-2Rα	0.8383	0.7937	0.7921	0.8028	1.2310
IP-10	0.8512	1.0627	1.4743	0.9660	1.5367
MIP-1β	0.8985	1.2883	1.0267	1.0257	0.9754
IL-8	0.9074	1.3195	1.0028	1.1780	0.9227
GROα	0.9337	1.0779	0.8543	0.9698	1.0296
CTACK	0.9474	0.9358	1.0033	0.9681	1.1286
MCP-1(MCAF)	0.9838	16.2556	0.9966	0.8086	0.8982
TNFβ	1.0135	0.8753	0.8152	0.6442	1.2496
IL-2	1.0324	0.9624	1.0730	1.3649	0.6001
IL-18	1.0660	1.1930	1.0340	1.0645	1.2438
IL-1α	1.0760	0.9523	0.9669	0.8696	1.5388
IL-1rα	1.1559	4.0214	1.6220	1.9585	0.7360
SCF	1.1584	1.1888	1.1956	1.1344	1.5535
PDGF-bb	1.1820	1.1830	1.3627	1.8366	1.1124
Eotaxin	1.1948	1.2337	1.3390	1.4456	1.6774
VEGF	1.2749	1.6210	1.9328	2.1658	1.2426
RANTES	1.2857	1.1759	1.7690	1.4019	1.4282
M-CSF	1.3299	1.5452	1.9018	1.2337	2.1465
IL-7	1.3316	1.6421	1.4309	1.5199	1.6575
MCP-3	1.3568	1.0830	1.2693	0.8602	2.0651
FGF basic	1.4055	1.4048	1.6151	1.8718	1.1152
GM-CSF	1.4679	1.5684	1.4339	1.2278	1.0226
SDF-1α	1.4845	1.4878	1.7933	1.9595	2.2563
sCD40L	1.4899	1.0377	2.1347	1.3364	2.5689
MIP-1α	1.5221	1.5882	1.6945	2.1876	1.1375
IL-33	1.5518	1.5232	1.6622	1.5858	2.2660
IL-17	1.7124	2.1088	2.4253	3.7954	1.2275
IL-17F	1.7802	1.4083	2.0998	1.7855	1.5024
IFNγ	1.8360	2.2328	2.2545	2.4735	2.0963
IL-9	1.9167	2.0734	1.8976	1.8670	1.4314
IL-1β	1.9201	2.2875	1.9894	2.2494	1.7892
IL-4	1.9378	2.5649	2.5050	2.7460	2.6155
TNFα	2.1990	2.9117	2.8939	3.2712	1.6760
TRAIL	2.2086	2.2952	2.6970	2.4267	3.0718
IL-13	2.3548	2.2645	2.0511	2.2400	2.5139
HGF	2.3870	1.4815	1.8309	1.5896	1.1896
IL-25	2.4868	1.7641	3.0086	1.8288	2.2186
G-CSF	2.5166	4.5924	2.8161	3.2957	2.7628
IL-10	2.5599	3.0066	3.1600	3.6470	2.9818
SCGFβ	2.6812	2.5675	2.8742	3.3780	2.0363
IL-5	2.6934	3.1685	3.3711	3.1084	8.9355
MIF	2.6944	1.8942	1.4160	1.4807	0.9236
LIF	2.8741	2.1537	2.8275	1.9227	6.6670
IL-22	2.9674	1.9497	2.9147	1.5633	2.4159
IL-23	3.4442	2.9422	4.7494	2.9133	3.4363
IL-12p70	4.0098	4.5469	5.0610	5.8253	4.7402
IL-21	5.2709	3.2876	6.7876	3.4594	5.3262
IL-31	6.2803	2.8972	5.9301	12.0710	10.9669

Fold change vs hospital control: 
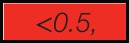

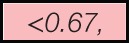

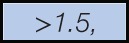

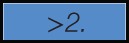

**Figure 3:**
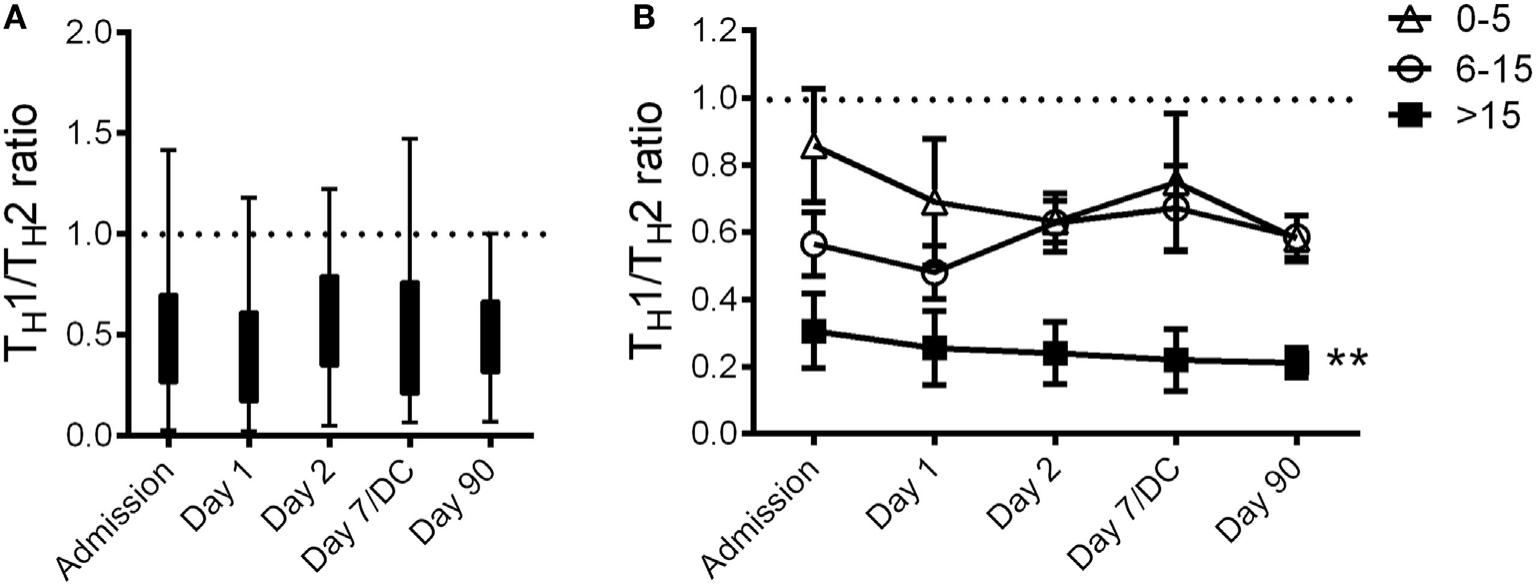
**Stroke-induced systemic shift toward T_H_2**. The plasma cytokine T_H_1/T_H_2 ratio of stroke patients at Admission (*n* = 36) and 1 day (*n* = 36), 2 days (*n* = 36), 7 days (or at discharge, DC; *n* = 14), and 90 days (*n* = 27) after stroke onset was presented in the box and whisker plot **(A)**. The plasma cytokine T_H_1/T_H_2 ratio of stroke patients with various National Institute of Health Stroke Scale at admission was also calculated **(B)**. Dotted line denotes Hospital controls. Error bars, SEM. **p < 0.01, one-way ANOVA with Bonferroni multiple comparisons *post hoc* test.

We focused our attention on the fold changes of four well-recognized T_H_1 (IFNγ, IL-2, IL-12, and TNFα) and T_H_2 cytokines (IL-4, 5, 10, and 13). For each stroke patient, we calculated their T_H_1/T_H_2 ratio by dividing the sum of the T_H_1 cytokine fold differences by the sum of the T_H_2 cytokine fold differences. We found that stroke significantly reduced the T_H_1/T_H_2 ratio in the blood of patients compared to their matching Hospital controls (Figure 3A; dotted line). In fact, this stroke-induced systemic immunological shift toward T_H_2 was evident throughout the observational period, including 3 months after the stroke onset (Figure 3A). Patients with severe strokes (NIHSS > 15) demonstrated a greater impairment in their systemic immunity as evidenced by the significant decrease of T_H_1/T_H_2 ratio as compared to the other stroke patients in this study (Figure 3B).

**Figure 4:**
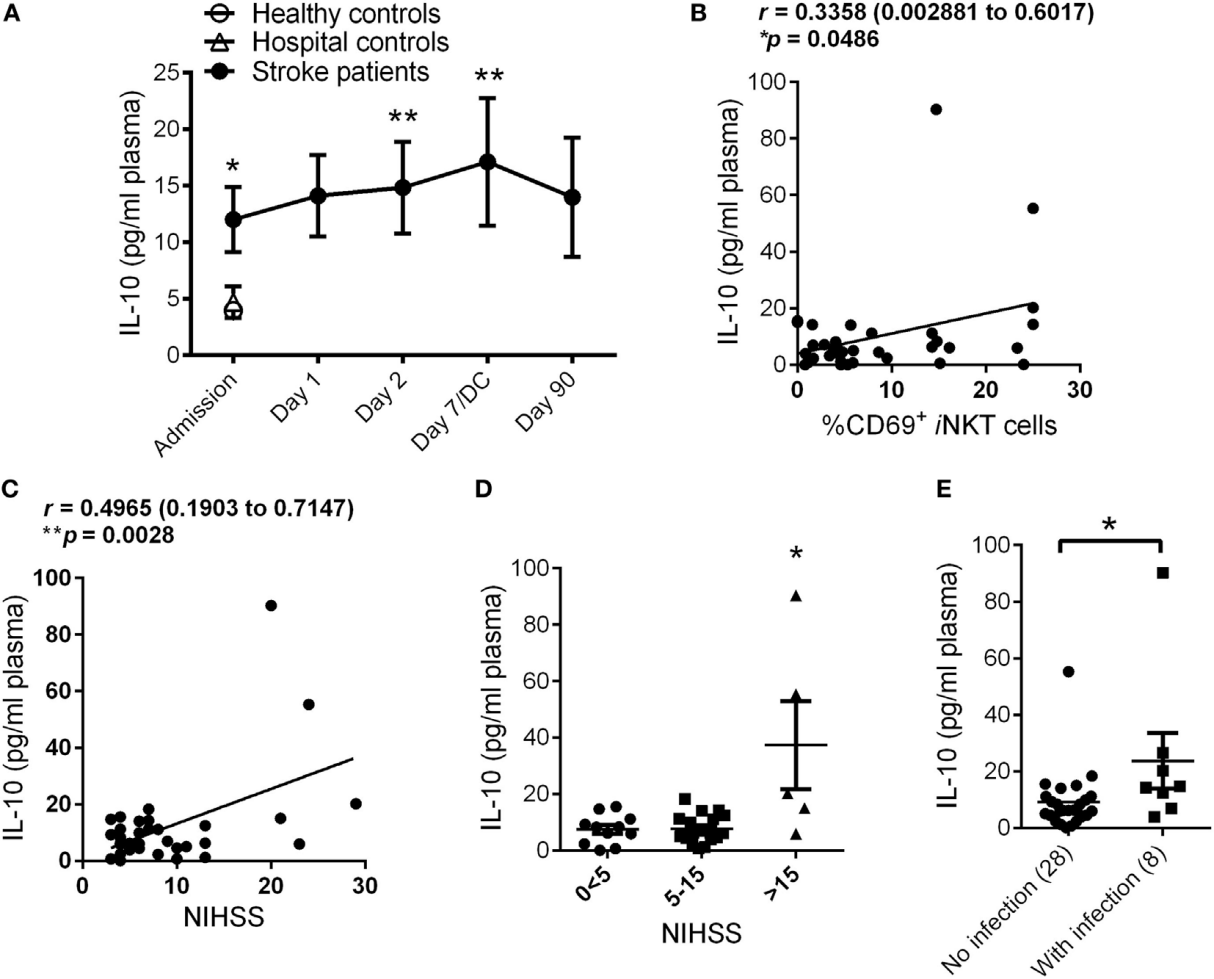
**Stroke-induced production of interleukin (IL)-10**. The plasma levels of IL-10 in Healthy (*n* = 10) and Hospital (*n* = 9) controls as well as stroke patients at Admission (*n* = 36) and 1 day (*n* = 36), 2 days (*n* = 36), 7 days (or at discharge, DC; *n* = 14), and 90 days (*n* = 27) after stroke onset were measured **(A)**. Error bars, SEM. **p < 0.01, *p < 0.05 vs Hospital controls, unpaired two-tailed Student’s *t*-test. Correlation coefficient was calculated between plasma IL-10 levels and invariant natural killer T (*i*NKT) cell activation **(B)** and National Institute of Health Stroke Scale (NIHSS) **(C)** at Admission. Pearson’s correlation coefficient (*r*) with 95% confidence interval expressed in brackets. Plasma level of IL-10 at Admission was measured in stroke patients with different NIHSS **(D)**. Error bars, SEM. *p < 0.05, one-way ANOVA with Kruskal–Wallis test. Plasma level of IL-10 at Admission was measured in stroke patients who later developed poststroke infection **(E)**. Error bars, SEM. *p < 0.05 vs no infection, unpaired two-tailed Mann–Whitney *U* test.

Within the group of T_H_2 cytokines, we found there was a significant increase of IL-10 production in patients at stroke onset compared to both Healthy and Hospital controls, and this stroke-induced elevation of IL-10 was observed for up to 3 months following the stroke event (Figure 4A), a trend that is very similar to the temporal profile of *i*NKT cell activation after stroke. We cannot confirm the increased peripheral IL-10 level after stroke was attributed by *i*NKT cell activation as that would require flow cytometric analysis of intracellular cytokine production of live *i*NKT cells. However, we found a positive correlation between plasma levels of IL-10 and *i*NKT cell activation at stroke onset (Figure 4B), suggesting that the production of IL-10 in stroke patients is likely to associate with the activation of peripheral *i*NKT cell. In addition, we also examined whether stroke severity is a key determinant of IL-10 level in patients. Indeed, there was a significant positive correlation between plasma levels of IL-10 and stroke severity (Figure 4C), with IL-10 levels in stroke patients highest when they were admitted to the hospital with a NIHSS above 15 (Figure 4D). We next examined whether plasma levels of IL-10 at Admission in stroke patients is a potential predictor of infection development. In spite of a relatively small cohort of patients, we found stroke patients who later developed infections showed significantly higher plasma level of IL-10 within 24 h of stroke onset (Figure 4E), this is in spite of NIHSS severity score (3, 2, and 2 infected patients with NIHSS subgroups 0 < 5, 5–15, and >15, respectively).

## Discussion

Stroke is one of the leading contributors to morbidity and mortality worldwide. Despite its recognized debilitating neurological deficits, the major cause of death in the post-acute phase after stroke is infection (1). These infections have traditionally been associated with precipitants such as aspiration, indwelling catheters, immobility related to severe strokes, or other health co-morbidities. Although such factors may play a role, it is now being increasingly recognized that stroke results in the impairment of the immune system (3). In our previous work in an experimental model of ischemic stroke, we demonstrated that stroke alters the function of *i*NKT cells, which are critical in the regulation of antibacterial defense (10). In addition, we found that reversing the stroke-induced impairment of *i*NKT cells restored normal function of the immune system and reduced poststroke infection, thus providing a novel avenue for limiting the major cause of death in stroke patients. To date, there is no information on the functional status of human *i*NKT cells after stroke. This knowledge is essential as we progress to develop potential *i*NKT cell-targeting novel agents to limit infection incidence in patients with stroke. Therefore, in this study, we performed a temporal analysis of *i*NKT cell activation and cytokine response in the blood of stroke patients and examine the association of these responses with stroke severity and susceptibility to poststroke infections.

Unlike animal models of stroke where we can control the location, duration, and severity of brain injury, clinical stroke studies consist of a much more diverse set of patient characteristics and stroke parameters. Therefore, variation between patients can mask distinctive differences. Often, this patient to patient variability can be controlled for by relating values to individual baseline data sets. With regards to this study, the inability to prospectively enroll patients prior to the stroke event made it impossible to gather pre-injury data for patients included in this study. Instead, we took the approach to use Hospital controls as a baseline to examine immune changes and calculate the fold differences of inflammatory mediators in individual stroke patients. Despite the relatively small patient sample size, which was likely to be limited by strict inclusion/exclusion patient enrollment criteria in this pilot prospective study, we demonstrated patients with stroke have altered immune response from onset that prolonged to 3 months after injury.

We observed a threefold increase in the expression of the activation marker CD69 in circulating *i*NKT cells of stroke patients compared to both matching Healthy and Hospital controls. These data suggest that the *i*NKT cell activation is specifically mediated by the brain injury induced by ischemic stroke and is independent of other causes of hospital admission. The magnitude of stroke-induced activation of *i*NKT cells was closely associated with the severity of stroke injury, with significant elevation reported in patients with an NIHSS of 15 or above. We proposed that stroke-induced activation of *i*NKT cells mediate an immunosuppressive response, *via* the release of IL-10, and render the patient with greater susceptibility to infection. Indeed, *i*NKT cell activation was positively correlated with IL-10 production in patients with stroke, and this rapid and sustained elevation of plasma IL-10 was exacerbated in patients who developed infections after stroke. IL-10 is a pleiotropic cytokine with important immunoregulatory functions. Early studies established that IL-10 limits cytokine and chemokine production from macrophages and dendritic cells during an inflammatory response (16). While localized IL-10 production in the brain is neuroprotective after stroke (17), we and others have found that peripheral levels of IL-10 are closely correlated with worsening of stroke outcome (7). Therefore, our data support the notion that IL-10 production after stroke is critical in the impairment of host antibacterial defense and suppression of the systemic immune system, potentially *via* a mechanism that involved the activation of *i*NKT cells. However, the confirmation that the increased peripheral IL-10 level after stroke was attributed by *i*NKT cells will require flow cytometric analysis of intracellular cytokine production of live *i*NKT cells in future studies. Nonetheless, the plasma level of IL-10 was significantly higher in patients with stroke who later developed infections, potentially suggesting the circulatory level of IL-10 at stroke onset has the potential to serve as a predictive biomarker for poststroke infection.

We examined the levels of a large panel of inflammatory mediators, which allow us to observe the systemic shift of immune profile after stroke. The stroke-induced shift of immunity toward a T_H_2-type occurred very rapidly as evidenced by significant reduction of T_H_1/T_H_2 cytokine ratio in stroke patients at the time of hospital admission, and this degree of T_H_2-skewed systemic immunity was dependent on stroke severity. In fact, this reduced T_H_1/T_H_2 ratio remained significantly lowered compared to Hospital controls for the duration of the study, suggesting a long-lasting shift of systemic immunity from T_H_1-type (cell-mediated “pro-inflammatory”) to a T_H_2-type (humoral “immunosuppressive”), and that there is limited resetting of immune barometer back to homeostasis after stroke. The results from this pilot study clearly point to a strong correlation between stroke severity, peripheral *i*NKT cell activation, IL-10 production, and severe immunosuppression. Importantly, these poststroke immunomodulatory effects continue long after the stroke event and are evident in patients after 3 months following the initial ischemic stroke onset.

In this study, we did not observe significant activation of circulating *i*NKT cells in stroke patients who later developed infection. Due to the fact that only five patients were analyzed in the severe stroke subgroup in this cohort (NIHSS > 15), thus interpretation of the current data should be handled with more caution to avoid over-interpretation regarding causality of circulating activated *i*NKTs on immune depression and infection in stroke patients. This is crucial to provide support to the suggestion that activated *i*NKT cells play a causal role in poststroke immune supression and infection after stroke. Nonetheless, our findings suggest that the higher activation of *i*NKT cells was the result of larger strokes, and future studies with larger cohort of patients will potentially unravel a causality relationship.

Damage to the brain after stroke is now known to be a largely immune mediated event not restricted to the cerebral. Stroke-induced systemic immune changes are believed to be a defensive mechanism of the brain with an attempt to lessen further cerebral damage; however, this renders the host prone to infections. A recent focus has been centered on reducing poststroke infection to improve clinical outcomes and patient health. Much attention has been drawn to preventive antibiotic treatment, though the majority of clinical studies have swayed against prophylactic antibiotic administration. Additionally, with the growing emergence of antibiotic-resistant pathogens, there is a clear need for alternative therapies to reduce infections after stroke. Immunomodulation therapies that augment the immune system to combat infections is an attractive avenue; however, caution is needed to ensure further cerebral injury is avoided. Future studies with larger patient cohorts should focus on the precise mechanisms of immune suppression after stroke in order to unveil alternative therapeutic targets to reduce stroke-associated infections.

## Ethics Statement

This study was carried out in accordance with the recommendations of CHREB at the University of Calgary with written informed consent from all subjects. All subjects gave written informed consent in accordance with the Declaration of Helsinki. The protocol was approved by the CHREB.

## Author Contributions

CW, MH, and PK conceived, designed, and supervised the project. CW, CJ, PT, and CL performed the experiments. CL was instrumental in the preparation of ethics application. AV, KR, and MH recruited and enrolled the patients for the study. CW, MH, and PK wrote the manuscript.

## Conflict of Interest Statement

The authors declare that the research was conducted in the absence of any commercial or financial relationships that could be construed as a potential conflict of interest.
